# The properties of transparent TiO_2_ films for Schottky photodetector

**DOI:** 10.1016/j.dib.2017.05.033

**Published:** 2017-05-23

**Authors:** Sung-Ho Park, Joondong Kim

**Affiliations:** Department of Electrical Engineering, Incheon National University, 119 Academy Rd., Yeonsu, Incheon 406772, South Korea

**Keywords:** Transparent Schottky devices, Photodetector, TiO_2_ film, Rapid thermal process

## Abstract

In this data, the properties of transparent TiO_2_ film for Schottky photodetector are presented for the research article, entitled as “High-performing transparent photodetectors based on Schottky contacts” (Patel et al., 2017) [1]. The transparent photoelectric device was demonstrated by using various Schottky metals, such as Cu, Mo and Ni. This article mainly shows the optical transmittance of the Ni-transparent Schottky photodetector, analyzed by the energy dispersive spectroscopy and interfacial TEM images for transparency to observe the interface between NiO and TiO_2_ film. The observation and analyses clearly show that no pinhole formation in the TiO_2_ film by Ni diffusion. The rapid thermal process is an effective way to form the quality TiO_2_ film formation without degradation, such as pinholes (Qiu et al., 2015) [2]. This thermal process may apply to form functional metal oxide layers for solar cells and photodetectors.

**Specifications Table**

**Table:** 

Subject area	*Electrical Engineering*
More specific subject area	*Photodetectors, Solar cells*
Type of data	*Figures*
How data was acquired	Field-emission transmission electron microscope (FETEM, JEOL, JEM-2100F) and energy dispersive spectroscopy (EDS). UV-visible spectrophotometer (Shimadzu, UV-1800)
Data format	*Raw, analyzed*
Experimental factors	*The optical profile and the interfaces of the transparent Schottky device* *(NiO/TiO*_*2*_*/FTO) were analyzed.*
Experimental features	*Pure Ti film formed a quality TiO*_*2*_*film by rapid thermal process.*
Data source location	*Incheon National University, Incheon 406772, Korea*
Data accessibility	*The data are with this article*

**Value of the data**

• The data presents the transparent Schottky contact for the photodetector device.
• Rapid thermal process is effective to form the quality TiO_2_ film without forming pinholes inside the layer [1].
• Elemental analyses clearly show the abrupt junction formation through the interfaces.
• The data is useful to design for transparent photoelectric device applications, including solar cells, photodetectors, and transparent semiconductor fabrications.

## 1 Data

**Fig. 1:**
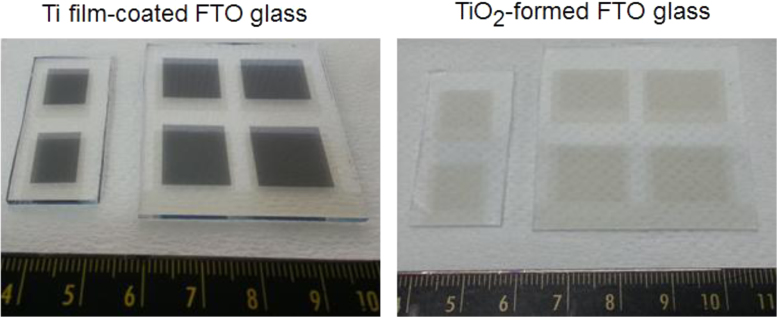
Photograph images of Ti film-coated FTO glass and TiO_2_-formed FTO glass. The RTP procedure was performed to transform the pure Ti film to the TiO_2_ film.

**Fig. 2:**
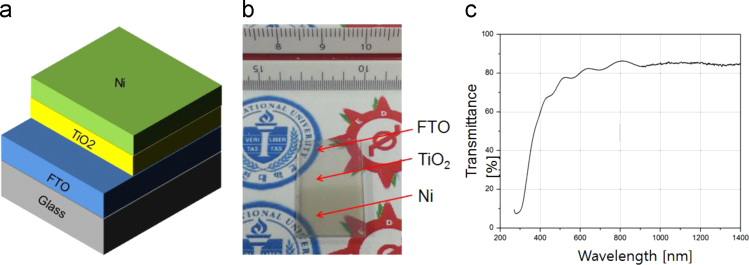
Transparent photodetector (Ni/TiO_2_/FTO/Glass) for (a) Schematics, (b) Photograph image, and (c) Transmittance profiles.

**Fig. 3:**
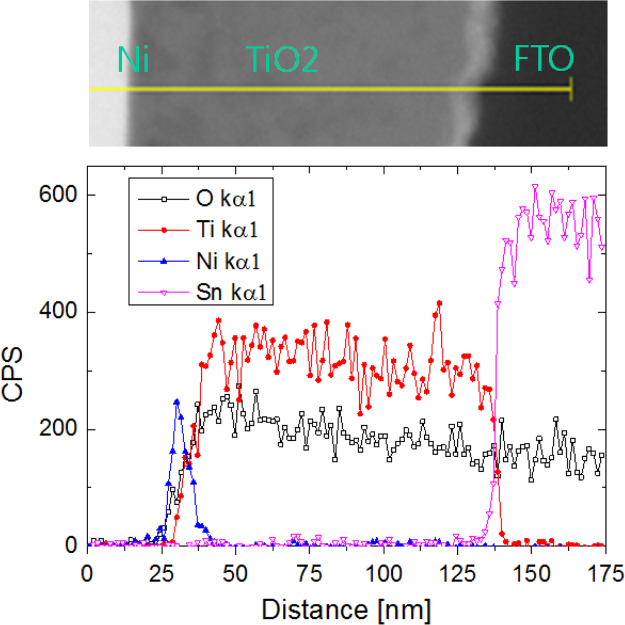
Cross-sectional TEM image and elemental line mapping using energy dispersive spectroscopy of Ni/TiO_2_/FTO layers.

**Fig. 4:**
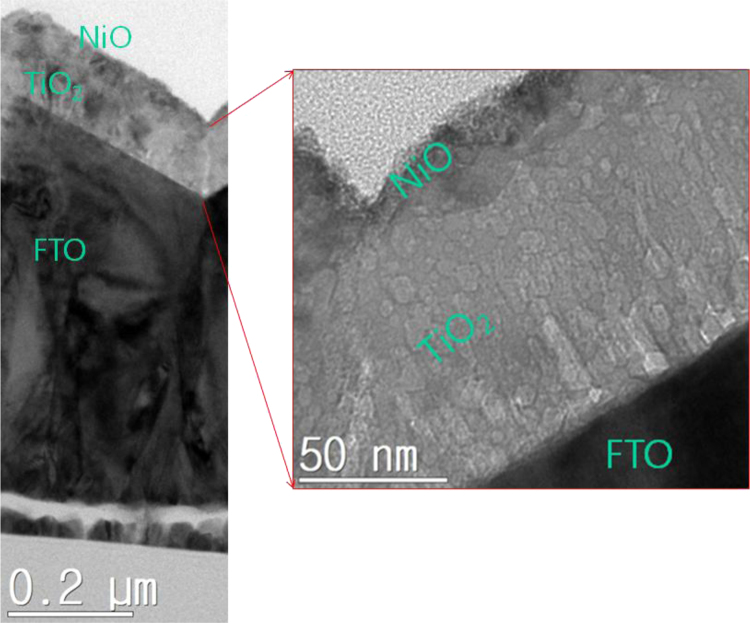
High-resolution TEM image of the NiO/TiO_2_/FTO layers. No pinholes were observed from the TiO_2_ film.

The datasets were acquired from the Schottky device of a thin Ni layer onto the TiO_2_ film, which is a route for high-performing transparent photoelectric devices. The fluorine doped-Tin oxide (FTO) glass was used as a substrate, where FTO layer serves as a transparent conductor. A quality TiO_2_ film was grown by rapid thermal process (RTP). Pure Ti film was initially coated on the FTO layer by sputtering method with 300 W onto a 4-in. Ti target (99.99%, iTASCO). After then RTP procedure was applied to transform TiO_2_ film at 700 °C for 10 min to ensure the transparency of Schottky type photodetector (Metal film/TiO_2_/FTO/glass). Various metal oxide films were formed by using different metal species, such as Cu, Mo and Ni. In order to investigate the stability of TiO_2_ film, the fast diffusion Ni metal was studied for the phenomenon of intrusion into TiO_2_ film. Fig. 1 and Fig. 2 are provided for the configuration of the transparent Schottky devices (Ni/TiO_2_/FTO/glass). The optical property was presented in Table 1. Fig. 3, Fig. 4 give the quality of TiO_2_ film and interfaces. By applying the RTP process, there is no serious degradation of TiO_2_ layer without pinholes, different from the e-beam evaporation method [2].

**Table 1:** Transmittance profiles of the transparent photodetector of NiO/TiO_2_/FTO/Glass.

Wavelength (λ)	λ>380 nm	λ>600 nm	λ>780 nm	380≤λ≤780
Transmittance [%]	81.6	84.3	84.8	76.6

## 2 Experimental design, materials and methods

### 2.1 Measurements

High-performing transparent Schottky photodetector was fabricated [1]. In order to observe the interfaces of the Schottky device (NiO/TiO2/FTO/Glass), a field-emission transmission electron microscope (FETEM, JEOL, JEM-2100F) was used. The TEM samples were prepared using a focused ion beam system (FIB, FEI, Quanta 3D FEG). *The* elemental compositions as line profile in the cross section of the transparent Schottky photodetector (NiO/TiO_2_/FTO/Glass) were determined by an energy dispersive spectroscopy (EDS) attachment to the FETEM. Optical characterization was carried out using a UV-visible spectrophotometer (Shimadzu, UV-1800) by recording the transmission of the transparent Shcottky device in the range 300–1400 nm.
