# Supplementary material for: The properties of transparent TiO2 films for Schottky photodetector
Source: Data Brief. 2017 May 23;13:171–4. doi: 10.1016/j.dib.2017.05.033 (PMC5454125; doi:10.1016/j.dib.2017.05.033)
Supplement: Supplementary file 1 — Supplementary material [file mmc1.docx]

***Conflicts of Interest Statement***

Re: DIB-D-17-00273

Title: The properties of transparent TiO_2_ films for Schottky Photodetector

We declare that this manuscript is original, has not been reported before, and is not currently being considered elsewhere. We also confirm that there is no known conflict of interest regarding this manuscript and its publication. The manuscript has been approved by all named authors.

Sincerely yours,


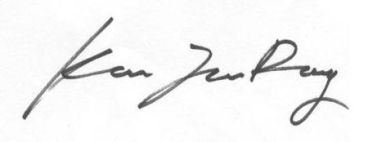


Joondong Kim

Joondong Kim, Ph.D./Professor

Department of Electrical Engineering,

Incheon National University

E-mail: joonkim@ incheon.ac.kr

Phone: +82-32-835-8770; fax: +82-32-835-0773
